# Protective effect of ghrelin in oxidative stress-induced age-related macular degeneration in vitro and in vivo

**DOI:** 10.1186/s10020-024-00920-w

**Published:** 2024-09-09

**Authors:** Jie Bai, Yanqing Wang, Yanze Li, Yan Liu, Shan Wang

**Affiliations:** 1 Department of Ophthalmology, the Fourth Affiliated Hospital of School of Medicine, and International School of Medicine, International Institutes of Medicine, Zhejiang University, Yiwu, 322000 Zhejiang P. R. China; 2 Department of Ophthalmology and Otorhinolaryngology, Yiwu Second People’s Hospital, Yiwu, 322000 Zhejiang P. R. China; 3 Department of Oral Pathology, School of Stomatology, Hainan Medical University, Haikou, 571199 P. R. China; 4 Department of Oral Pathology, School of Stomatology, Hainan Medical College, Haikou, 571199 P. R. China

**Keywords:** Ghrelin, Oxidative stress, Age-related macular degeneration, Vitro and vivo

## Abstract

Oxidative damage to human retinal pigment epithelial (RPE) cells is the main cause of age-related macular degeneration (AMD), in our previous work, we showed that ghrelin has an antioxidative effect on human lens epithelium (HLE) cells, however, the studies of using ghrelin in treating the degenerative diseases of the retina have rarely been reported. In this article, we assessed the effect of ghrelin on preventing oxidative stress induced by hydrogen peroxide (H_2_O_2_) in ARPE-19 cells and its mechanism. We observed that pretreatment with ghrelin protected ARPE-19 cells from H_2_O_2_-induced cell oxidative injuries and apoptosis responses. Furthermore, an oxidative stress-induced mouse model of AMD was established via injection of sodium iodate (NaIO_3_) to tail veins, and treatment with ghrelin preserved retinal function, and protected photoreceptors.

## Highlights

The first to report ghrelin’ protective effect on H_2_O_2_-induced RPE cells.

Treatment with ghrelin inhibits NaIO_3_-induced retinal cell apoptosis in vivo.

Combined with in vitro and in vivo experiments, the results are comprehensive and reliable.

## Introduction

Age-related macular degeneration (AMD) is the main cause of vision decline among the elderly in China (Gao et al. 2018). Retinal pigment epithelial (RPE) cells located between photoreceptors and choroidal capillaries in the retina are believed to be involved in the occurrence of AMD (Deng et al. 2021a, b).

Oxidative stress and oxidative stress-induced cellular degeneration both play critical roles in AMD (Saito et al. 2018). Given its high level of oxygen consumption, continual exposure to light, and intensive oxygen metabolism, the retina is particularly susceptible to oxidative damage, and reactive oxygen species (ROS) are major factor involved in RPE cell death, which underlies AMD (Mano et al. 2021; Pinilla and Maneu 2022).

Antioxidant supplementation is a plausible strategy to avoid oxidative stress and thereby maintain retinal function (Rajapakse et al. 2017). Ghrelin is a 28-amino-acid peptide obtained from human or rat stomachs. Its biological activities include thermogenesis, improvement of neuron survival, regulation of apoptosis, anti-inflammation, immunosuppression, and antioxidation. Ghrelin is an effective drug against various diseases, including the occurrence of reperfusion arrythmias, gastrointestinal stromal tumours, diabetic neuropathy, myocardial infarction, and lung injury (Spiridon et al. 2021; Chen et al. 2019). In our previous work, we assessed the antioxidative effect of ghrelin on human lens epithelium (HLE) cells and proved that it may have a therapeutic effect on cataracts (Bai and Yang 2017).

In this study, we used H_2_O_2_ to simulate in vitro the oxidative stress microenvironment in ARPE-19 cells in vitro and investigated the anti-oxidative and anti-apoptotic features of ghrelin. We also conducted an in vivo study in which we constructed AMD rat models to demonstrate that ghrelin could decrease NaIO_3_-induced oxidative injury and could preserve retinal function to ultimately demonstrate the role of ghrelin in AMD.

## Materials and methods

### Reagents and antibodies

ARPE-19 cells were obtained from the American Type Culture Collection (Manassas, VA, United States). Ghrelin (99.5%) was purchased from Sigma-Aldrich (St. Louis, MO, United States), dissolved in dimethyl sulfoxide and stored (DMSO), and stored at − 20 °C for later use. 3-(4,5-Dimethylthiazol-2-yl)-2,5 diphenyl tetrazolium bromide (MTT) assays, hematoxylin and eosin (HE) staining and one-step TUNEL apoptosis assay kits were obtained from the Beyotime Institute of Technology (Shanghai, China). 2ʹ,7ʹ-Dichlorofluorescein diacetate (H_2_DCFDA) was obtained from Invitrogen (Carlsbad, CA, United States). Annexin V − fluorescein isothiocyanate (FITC)/propidium iodide (PI) was obtained from BD Biosciences (Mountain View, CA, United States). Superoxide dismutase (SOD) activity and catalase (CAT) kits were procured from Nanjing Jiancheng Bioengineering Institute (Nanjing, China), Bradford’s protein assay kit was obtained from the Beyotime Institute of Technology (Shanghai, China). Anti-HO-1, anti-nuclear factor erythroid 2related factor 2 (NFE2L2/NRF2), anti-Bcl-2, and anti-Bax antibodies were purchased from Santa Cruz Biotechnology (Santa Cruz, CA, United States).

### Cell culture

ARPE-19 cells (ATCC, United States) were maintained in Dulbecco’s Modified Eagle Medium (DMEM) supplemented with 10% foetal bovine serum, 50 mg/mL gentamicin and 300 mg/mL L-glutamine at 37 °C in 5% CO_2_ incubator. In this study, 300 µmol/L H_2_O_2_ was chosen as the final concentration to detect the cytotoxicity of H_2_O_2_. The cells were divided into four groups: a control group, 300 µmol/L H_2_O_2_ (oxidative damage) group, 300 µmol/L H_2_O_2_ + 10^− 7^ mol/L ghrelin group, and 300 µmol/L H_2_O_2_ + 10^− 6^ mol/L ghrelin group.

### MTT assay

Cells (1 × 10^4^/L) were seeded into 96-well plates and treated with ghrelin (10^− 10^–10^− 6^ mol/L) or with H_2_O_2_ (0–400 µM) for 24 h; cell viability was subsequently analysed. To test the effect of ghrelin following an oxidative stress, we treated the cells with ghrelin (10^− 7^ mol/L and 10^− 6^ mol/L) for 24 h and then exposed them to H_2_O_2_ (300 µM) for another 24 h; the culture medium was then refreshed, and 10 µL of MTT reagent (0.5 mg/mL) was added for 4 h. Subsequently, the wells were emptied, and then DMSO (150 µL) was added. The absorption values were measured at 490 nm using a microplate reader (BioTek, VT, United States).

### Apoptosis assay

The cell apoptosis rate was determined through double staining with FITC/PI. Cells were incubated with ghrelin (10^− 7^ mol/L and 10^− 6^ mol/L) for 24 h and then exposed to H_2_O_2_ (300 µmol/L) for 24 h. The cells were collected and then suspended in 400 µL binding buffer (containing 5 µL FITC and 5 µL PI) for 20 min. The cell apoptosis percentage was recorded and analysed by flow cytometry (BD Biosciences, San Diego, CA, United States).

### ROS assay

The cells were incubated with the fluorescent probe H_2_DCFDA (15 µM) in serum-free DMEM for 30 min at 37 °C in the dark. Then the cells were then resuspended in binding buffer and analysed by flow cytometry.

### SOD activity and CAT content

SOD activity and CAT content were assessed with a spectrophotometer. The cells were lysed with 0.05 mol/L Tris. HCl extraction buffer on ice, and cell lysates were used to assess SOD activity and CAT content, respectively. SOD activity was measured with the reaction mixture containing L-methionine (13 mM), riboflavin (75 µM), Na-EDTA (0.1 mM), sodium-phosphate buffer (pH 7.8; 50 mM), and enzyme extract (0.1–0.2 ml). Reaction was carried out in test tubes at 25 °C under fluorescent lamp (40 W). The absorbance was recorded at 560 nm. CAT content was measured with the reaction mixture containing H_2_O_2_ (65 mM), phosphate buffer (pH 7.8; 50 mM), and enzyme extract was incubated at 37 °C for 1 min. The OD240 was measured immediately at 25 °C.

### Western blot analysis

Cells were lysed on ice for 30 min, the concentrations of protein extracts was detected by Bradford’s protein assay kit. Proteins (30 mg) were loaded onto SDS-PAGE gel and then transferred onto nitrocellulose membrane blocked with 5% milk for 1 h at room temperature. Mouse anti-HO-1 polyclonal antibody (1:100, sc-390991), mouse anti-NFE2L2/NRF2 polyclonal antibody (1:100, sc-365949), mouse anti-Bax polyclonal antibody (1:100, sc-7480) and mouse anti-Bcl-2 polyclonal antibody (1:100, sc-71022) were used as primary antibodies in 5% milk at 4 °C overnight. Goat anti-mouse antibodies (1:10000, Zhongshan Golden Bridge, Guang Zhou, China) were used as secondary antibodies for 2 h at room temperature. The bands were developed using the enhanced chemiluminescence (ECL) detection bands intensities were detected and exposed to X-ray film, and the results were analysed using Quantity One version 4.62 software (Bio-Rad Laboratories, CA, United States).

### Animals and treatment

Adult male Sprague–Dawley rats (210–270 g) were obtained from the First Affiliated Hospital of Harbin Medical University (laboratory animal licence number: SCXK [Heilongjiang Province] 2019-012) and randomly divided into four groups: control group (*n* = 8), ghrelin group (*n* = 8), NaIO_3_ group (*n* = 8), and NaIO_3_ + ghrelin group (*n* = 8). The rats in the NaIO_3_ group were given NaIO_3_ (20 mg/kg) once by tail vein injection for 28 days, and the rats in NaIO_3_ + ghrelin group were given ghrelin (100 µg/kg) daily through subcutaneous injection for 14 days before being injected by a tail vein injection with NaIO_3_ for 28 days. Rats in the control group received a single intravenous injection of 0.9% NaCl. The animal experiments were approved by the Institutional Animal Care and Use Committee (IACUC) of Harbin Medical University. Fundus images were obtained using a retinal imaging system (Optomap Panoramic 200Tx, Optos company, United Kingdom).

### Preparation of retinal tissues

The rats were euthanized by dislocating their cervical spine. For retinal morphometric analysis, the eyeballs were enucleated, fixed in 4% paraformaldehyde at 4˚C for 2 h, washed with phosphate-buffered saline (PBS) and then embedded in paraffin. Sections were subsequently subjected to hematoxylin and eosin (H&E) staining and immunocytochemistry.

### HE staining

Retinal Sect. (5 μm) across the optic nerve head were mounted on slides and dyed with H&E at 37˚C for 5 min, rinsed with water; dehydrated with alcohol (100% alcohol for 5 min, 75% alcohol for 5 min, and 50% alcohol for 5 min), immersed in xylene, and embedded in paraffin. The sections were then stained in eosin for 5 min, xylene for 5 min, and mounted with resin. Images were captured by light microscope (200×). The thickness of total retina, inner nuclear layer (INL) and outer nuclear layer (ONL) were measured from three different rats from every group. Retinal thickness was determined as the distance between the RPE layer and the internal limiting membrane (ILM) of the retina. K-Viewer software (Ningbo Jiangfeng Biological Information Technology, China) was uesed to measure the thickness.

### Retinal TUNEL staining

Prepared retinal histological sections were also stained with TUNEL labelling using a one-step TUNEL apoptosis assay kit (Beyotime Institute of Biotechnology, Shanghai, China) to detect apoptosis in cells. Paraffin sections from histological assessment were routinely deparaffinized, rehydrated, and then rinsed with PBS. Sections were incubated with the TUNEL reaction in the dark mixture at 37˚C for 60 min and then rinsed with PBS. Sections were mounted with 4ʹ,6-diamidino-2-phenylindole (DAPI), and images were captured using an inverted fluorescent microscope. The number of apoptotic cells was counted in three sections of each eye and averaged.

### Transmission electron microscopy

Retinas were fixed with 2% glutaraldehyde and 2% glutaraldehyde in 0.1 M PBS at pH 7.4 for 24 h, stained with uranium acetate solution, dehydrated with ethanol–acetone gradient solution, and embedded in Epon. Ultrathin (80 nm) of pellets were stained with lead citrate and uranium acetate for 5 min and then observed under a transmission electron microscope (Hitachi High-Technologies Corporation, Tokyo, Japan).

### Statistical evaluation

All experiments were performed at least three times. The data were presented as the means ± standard deviation (SD). Two-tailed Student’s *t*-test and one‐way ANOVA were used for statistical analysis using Graph Pad Prism 6.0 software (GraphPad Software, Inc., United States).

## Results

### Ghrelin inhibited H2O2-induced ARPE-19 cells cytotoxicity

**Fig. 1:**
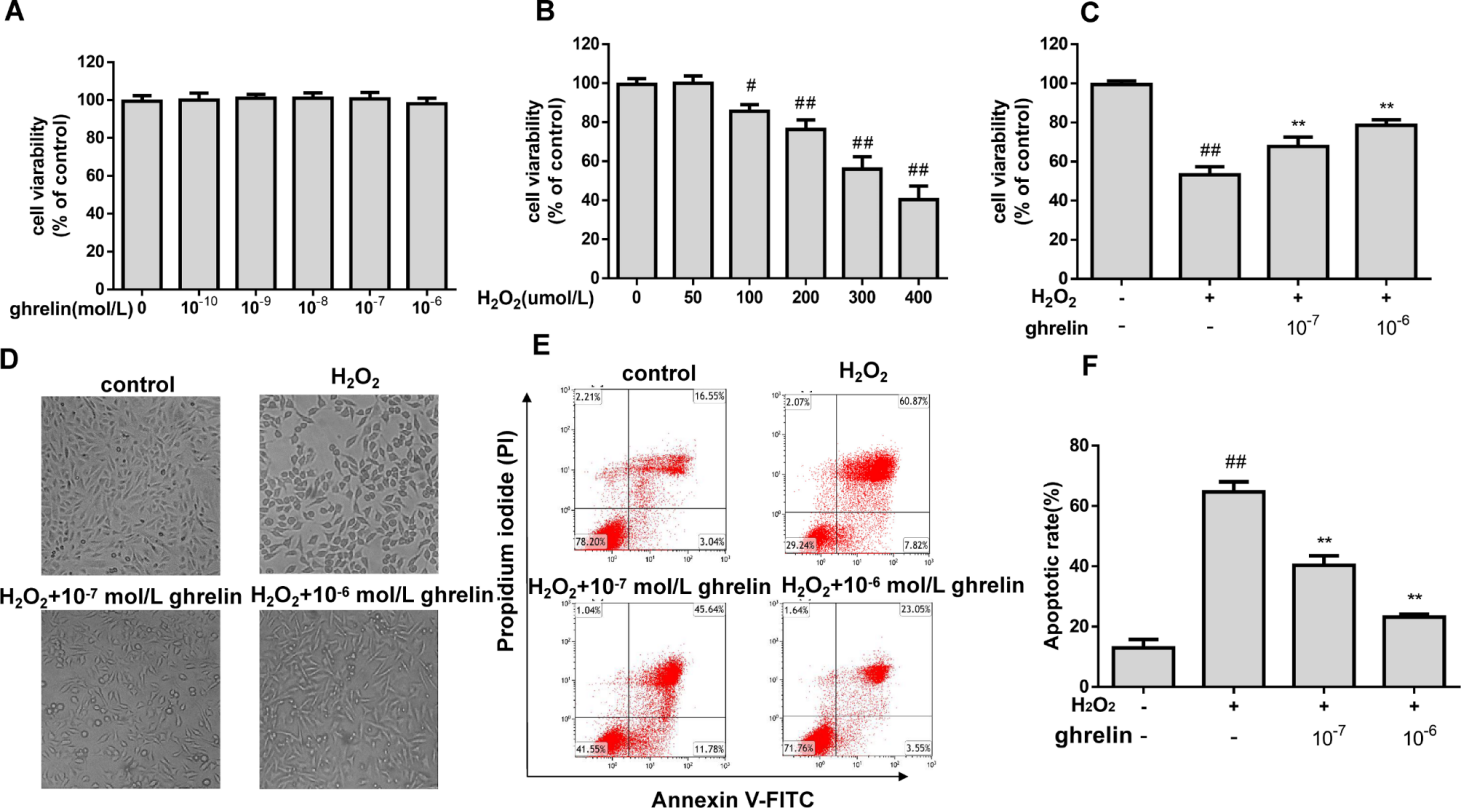
The effect of ghrelin on cell viability and apoptosis. (**A**) Cells were incubated with a series of concentrations of ghrelin (10^− 10^-10^− 6^ mol/L) for 24 h and then detected by MTT assay. (**B**) Cells were incubated with a series of concentrations of H_2_O_2_ (50–400 µmol/L) for 24 h. (**C**) Cells were incubated with ghrelin for 24 h and then exposed to H_2_O_2_ (300 µM) for 24 h. (**D**) Morphological images of ARPE-19 cells. (**E**) Cells apoptosis detected by flow cytometric analysis using double staining with FITC/PI. (**F**) Quantitative analyses of the apoptosis rate in ARPE-19 cells (*n* = 3). ^#^*P* < 0.05, ^##^*P* < 0.01, compared with the control group; **P < 0.01, compared with the oxidative damage group

To examine whether ghrelin is cytotoxic to ARPE-19 cells, we performed MTT assay on cells treated with a series of concentrations of ghrelin. The results showed that the concentrations 10^− 10^–10^− 6^ mol/L did not affect cell viability (Fig. 1A). The cytotoxicity of H_2_O_2_ on ARPE-19 cells was also evaluated. We detected that the cell viability gradually decreased after exposure to H_2_O_2_, and a 50% cytotoxicity index was found at 300 µM (Fig. 1B). Therefore, we chose 300 µM of H_2_O_2_ as the experimental concentration for the subsequent experiments. We also detected the protective effect of ghrelin on H_2_O_2_-induced ARPE-19 cells by MTT assay, as shown in Fig. 1C: ghrelin pretreatment prevented the loss of cell viability.

### Ghrelin treatments inhibits apoptosis in ARPE-19 cells

Morphological images of the ARPE-19 cells were detected using a phase contrast microscope. In the control group, the cells were long, spindle-shaped, and regular, whereas after H_2_O_2_ intervention, the space between the cells increased and the cell volume decreased, with some cells becoming spherical in shape (Fig. 1D). Cells in the Annexin V-FITC/PI results (Fig. 1E and F) indicated that exposure to H_2_O_2_ led to a significantly higher rate of apoptosis (64.72% ± 3.30%) compared with that of the control group (12.97% ± 2.78%). With the pre-treatment of ghrelin, the percentages of apoptotic cells decreased to 40.35% ± 3.15% and 23.20% ± 0.87%, respectively, indicating that ghrelin treatments inhibited apoptosis in ARPE-19 cells.

### Ghrelin reduced oxidative stress in ARPE-19 cells

**Fig. 2:**
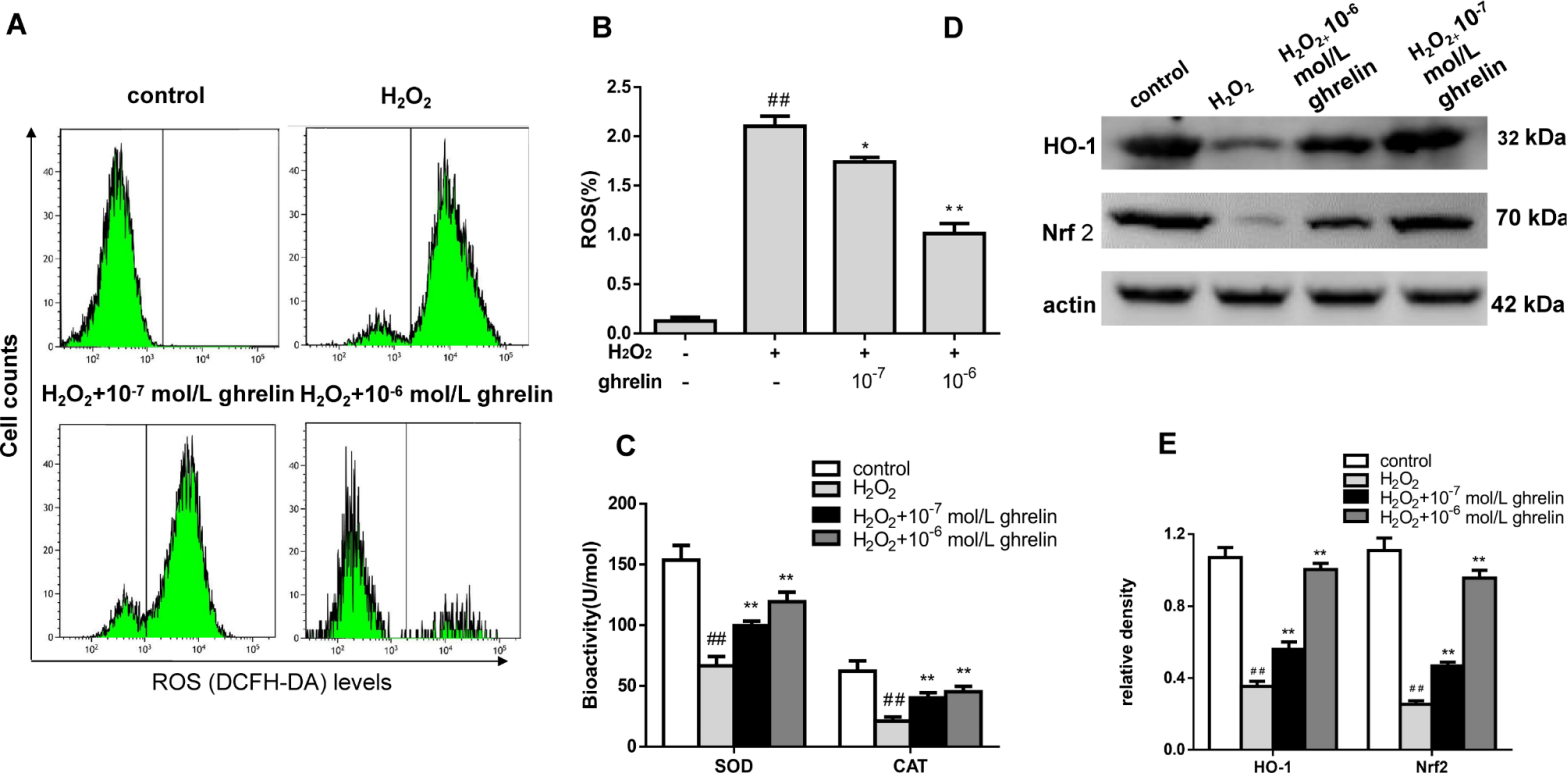
Ghrelin inhibited oxidative stress in ARPE-19 cells. (**A**) ROS levels were analysed by flow cytometry in the FITC-A channel. (**B**) Quantitative analyses of ROS generation. (**C**) Biochemical analyses revealed the alterations in SOD and CAT. (**D**) The expression levels of HO-1 and NRF2 in ARPE-19 cells detected by western blot. (**E**) Statistical analysis of western blot data (*n* = 3). ^##^*P* < 0.01, compared with the control group; *P < 0.05, **P < 0.01, compared with the oxidative damage group

As displayed in Fig. 2A and B, the level of ROS was higher in the oxidative damage group than that in the control group (*P* < 0.01), but it was decreased in the ghrelin-treated group (all *P* < 0.05). We also examined SOD activity and CAT content within cells; the results showed that H_2_O_2_ treatment led to an apparent decrease in SOD activity and CAT content, which could be reversed by treatment with ghrelin and were enhanced with an increase in the concentration of ghrelin (Fig. 2C). Levels of HO-1 and NFE2L2/NRF2 were examined by western blot to detect the antioxidative capability of ghrelin on H_2_O_2_-stimulated ARPE-19 cells. Notably, exposure to H_2_O_2_ decreased HO-1 and NFE2L2/NRF2 expression, relative to the control group. Pretreatment with ghrelin significantly enhanced the expression of HO-1 and NFE2L2/NRF2 compared with the oxidative damage group (Fig. 2D and E).

### Effect of ghrelin on the retina

**Fig. 3:**
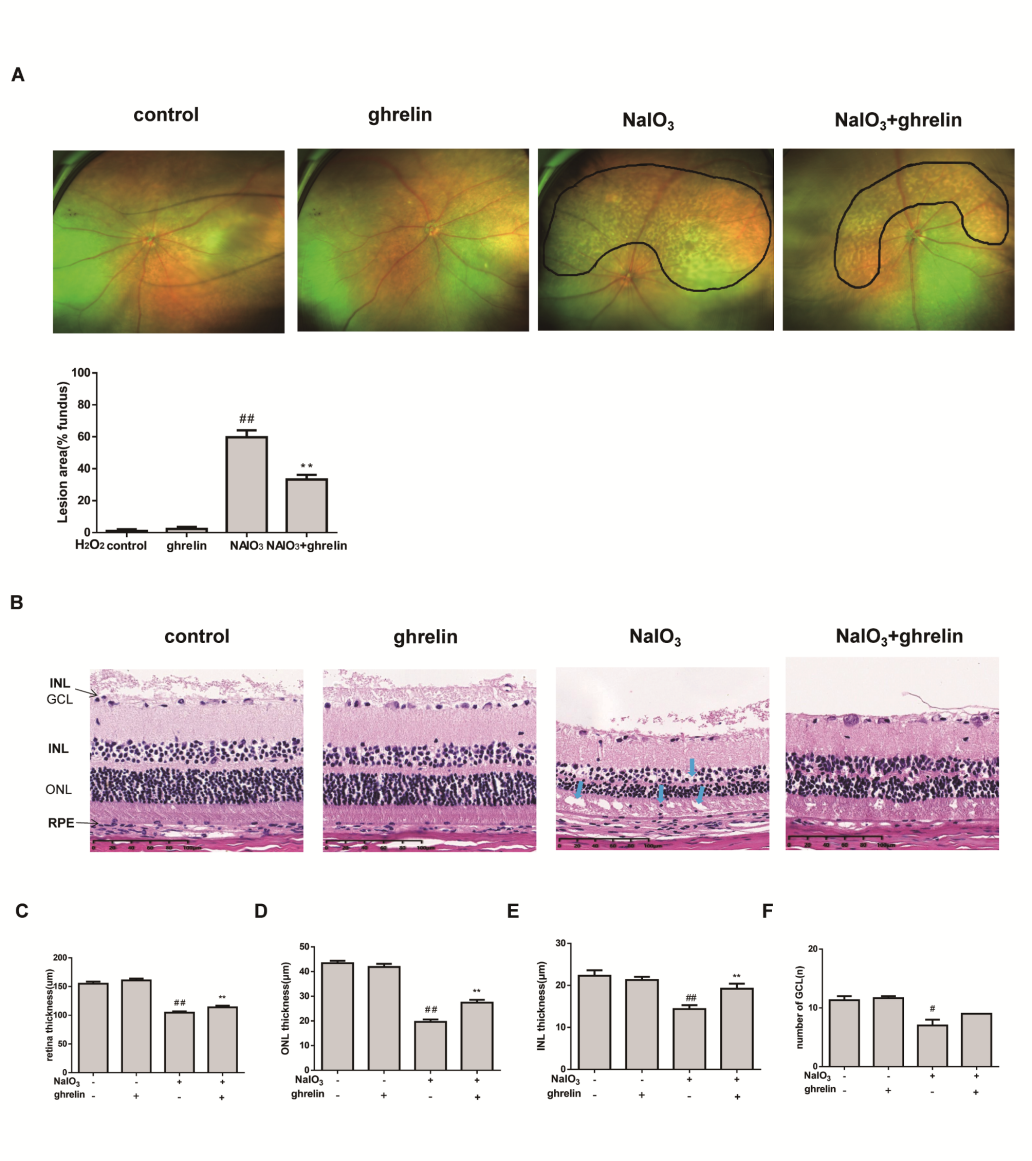
Effects of ghrelin on retinal histology. (**A**) Colour fundus photography was observed using a retinal imaging system. Area in black circle indicated the retina damages caused by NaIO3. (**B**) quantification of lesion area per fundus image for each group. (**C**) Retinal structure stained with H&E (200×). (**D**) Corresponding quantitative analysis of retinal thickness from the pigment epithelium to the inner limiting membrane. (**E**) Quantitative assessment of the thickness of the ONL. (**F**) Quantitative assessment of the thickness of the INL. (**G**) The number of retinal ganglion cells. ^#^*P* < 0.05, ^##^*P* < 0.01, compared with the control group; **P < 0.01, compared with the oxidative damage group

Large areas of retinal degeneration were seen in the NaIO_3_ group, and ghrelin treatment decreased the area of yellowish white wart-like deposits. We valved the area of yellowish white wart-like deposits in retina by color fundus photography, there were no obvious yellowish white wart-like deposits in control group and ghrelin group, but in NaIO_3_ group, the area enlarged obviously (we circled this area by black line), and in NaIO_3_ + ghrelin group, the area of yellowish white wart-like decreased, Fundus image of NaIO_3_ + ghrelin group showed remarkably smaller lesion areas (∼ 22% of reduction) compared with NaIO_3_ group (Fig. 3A and B). As shown in Fig. 3C and D, the thickness of the overall retina in the ghrelin group was almost the same as that of the control group. In contrast, NaIO_3_ injection led to a reduction in mean retinal thickness of approximately 27%, and a thinner INL and ONL were seen in the NaIO_3_ group (Fig. 3E and F). An increase in retinal thickness was found in the NaIO_3_ + ghrelin group. Retina layers in control group and ghrelin group were organized and cellular stratification neat, however, in NaIO_3_ group, the cells in the INL and ONL became smaller, cells arrangement were disordered and loosed, cells arrangement and morphology in NaIO_3_ + ghrelin group become better than that in NaIO_3_ group.

We also found that the retinal ganglion cell number was also decreased by NaIO_3_ injection (a reduction of approximately 57%) compared with the control group (Fig. 3F), which indicates that NaIO_3_ caused structural damage to the retina, but the detrimental effect of NaIO_3_ was alleviated by ghrelin treatment.

### Ghrelin inhibits NaIO3-induced retinal cell apoptosis in vivo

**Fig. 4:**
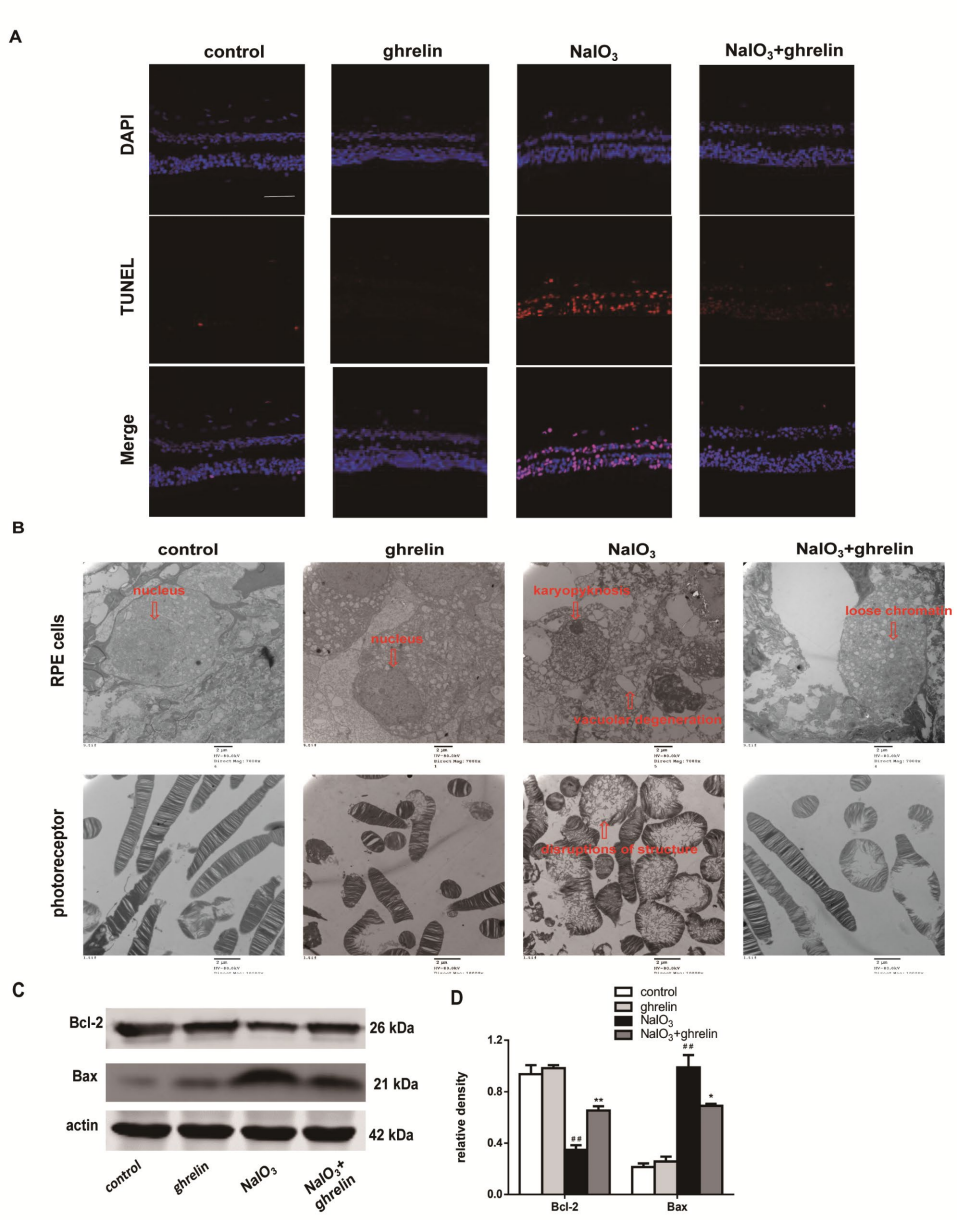
(**A**) A one-step TUNEL apoptosis assay kit was used to detect apoptotic cells. DAPI (blue) was used to stain the nuclei, and TUNEL (red) was used to stain apoptotic cells (scale bar: 100 μm). Red arrows indicated TUNEL-positive cells. (**B**) Morphologic changes in ARPE-19 cells and photoreceptors detected by transmission electron microscopy. (**C**) The expression levels of Bcl-2 and Bax in retinas were detected by western blot. (**D**) Statistical analysis of western blot data (*n* = 3). ^##^*P* < 0.01, compared with the control group; *P < 0.05, **P < 0.01, compared with the NaIO_3_ group

To follow the process of RPE-thickness recovery, we next investigated photoreceptor cell degeneration in the retinas. As shown in Fig. 4A, almost no apoptotic cells were found in the control group and ghrelin group, and after NaIO_3_ injection, the number of TUNEL-positive cells (red) clearly increased in the outer nuclear layers (ONL), indicating that the cells were undergoing apoptosis. However, in the NaIO_3_ + ghrelin group, the number of TUNEL-positive cells decreased, indicating that ghrelin may attenuate photoreceptor loss in the retina and preserve the structure of the retina.

### Cell ultrastructure observation detected by transmission electron microscopy

Cells in the control group and the ghrelin group exhibited complete cell forms; in the NaIO_3_ group, the RPE cells’ nuclear membranes were incomplete, a large number of lipid droplets and cytoplasmic vacuoles were seen, and photoreceptors were ruptured. The transmission electron microscopy results also showed degeneration of the photoreceptor layer of the retina after the induction of NaIO_3_, the photoreceptor cells were swollen and ruptured and lost their complete structure. In the ghrelin treated group, the cells’ morphologies were further improved (Fig. 4B).

### Ghrelin protects the retina by reducing apoptosis

Compared with the retina without any treatment, NaIO_3_**-**induced retina had down-regulation of Bcl-2 and up-regulation of Bax. These results indicated that ROS production was related to apoptosis of the retina induced by NaIO_3_. However, ghrelin resulted in increased Bcl-2 expression and reduced Bax expression (Fig. 4C and D), which indicated that ghrelin protected the retina by regulating the apoptosis pathway.

## Discussion

Oxidative stress from the environment could cause various damages including RPE cell death, DNA damage, and destruction of membrane integrity. The generation of ROS has been considered to be a major factor in ageing and disease (Deng et al. 2021a, b; Chang et al. 2022). AMD, and cataract, are degeneration diseases that accompany aging and are responsible for vision impairment and blindness. Hence, the development of effective drugs for macular degeneration to improve AMD debilitating visual disease is an urgent issue. Regulation of ROS levels is a key index for maintaining cell function. Studies have shown that antioxidant supplements have beneficial effects on eye diseases, preventing or even improving both AMD and cataract (Li et al. 2021a, b, 2022; Kumar 2022).

Oxidative stress is known to contribute to the development of AMD. Exposure to H_2_O_2_ is a common model that is used to evaluate oxidative stress susceptibility and antioxidant activity of RPE cells (Henning et al. 2022; Chen et al. 2022). Excessive ROS may cause significant damage to RPE cell structures and functions (Srivastava et al. 2022; You et al. 2021). Many studies have found that ROS generated by H_2_O_2_ lead to photoreceptor cell death and RPE cells apoptosis, it is similar to the pathogenesis of AMD (Xie et al. 2022; Zhang et al. 2022). In the present study, a high level of endogenous ROS is highly correlated with RPE cells death induced by H_2_O_2_, and it was effectively blocked by ghrelin treatment.

Ghrelin has several advantages, including the ability to reduce the oxidative stress, rescue lung damage, and upregulate the expression of Sirt1, PGC-1a, and UCP2 after hypoxic-ischemic encephalopathy (Davies 2022; Bai et al. 2021). The protective effect of ghrelin in the eye has been reported in several studies. Many studies have shown that ghrelin can pass through the blood–eye barrier and it is safe for use in the eye (Can et al. 2015; Rocha-Sousa 2014). Liu S. et al. have shown that ghrelin can improve retinal ganglion cell survival in rat models with Parkinson’s disease by AKT-mTOR signalling (Liu et al. 2018). Zhu K. et al. showed that ghrelin can provide a neuroprotective effect in chronic intraocular hypertension model retina, which indicates that ghrelin may be a potential treatment for glaucoma (Zhu et al. 2017). Our previous work also demonstrated that ghrelin suppresses ROS production and cell apoptosis in HLE cells and rat lens (Bai and Yang 2017); based on the fact that it is safe to use in the eye and the protective effect it has on various ocular cell tissues, we further explored the effect of ghrelin on ARPE-19 cells and retina. Different from our previous work, the present study is the first to test the protective effect of ghrelin in ARPE-19 cells. On the basis of the methods used before, we use H_2_DCFDA staining through flow cytometry to detect ROS levels within cells, transmission electron microscopy to detect cell morphologic changes, and in vivo experiments (H&E and TUNEL staining) to detect changes in the retina. These results further confirmed the protective effect of ghrelin on ocular tissues (lens and retina) and provided a theoretical basis for its application.

In our study, we found that ROS levels in ARPE-19 cells increased significantly in response to oxidative stress; however, ghrelin reversed this phenomenon. Furthermore, ghrelin pretreatment significantly increased the activity of SOD and CAT which were suppressed by H_2_O_2_. Intracellular redox status, which is determined by the balance between ROS and antioxidant defence mechanism, is the key facto in influencing programmed cell death. NFE2L2/NRF2 is a regulator of ROS and a sensor of oxidative stress, and it could regulate the ROS homeostasis in many cell types (Li et al. 2021a, b). Our results showed that ghrelin dose dependently activated the expression of NFE2L2/NRF2. These results support that ROS-mediated oxidative stress leading to ARPE-19 cells damage, and ghrelin pretreatment could play an antioxidant role by activating NFE2L2/NRF2 signalling pathways (Kumar and Mandal 2018).

Bcl-2 and Bax are members of the Bcl-2 family, which is involved in the regulation of mitochondrial permeability. Bcl-2 is an inhibitor of apoptosis gene. Bax not only promotes apoptosis, but also antagonizes the inhibitory effect of Bcl-2 on apoptosis. Bax can form oligomers, transfer from the cytoplasm to the mitochondrial membrane, form a polymer with Bcl-2, enhance the permeability of mitochondria, and finally lead to the release of cytochrome c. Therefore, the increase in Bcl-2 and the decrease in Bax levels indicate that the resistance of cells to apoptosis is enhanced, and vice versa, which is the sign of cells being protected by drugs.

**Fig. 5:**
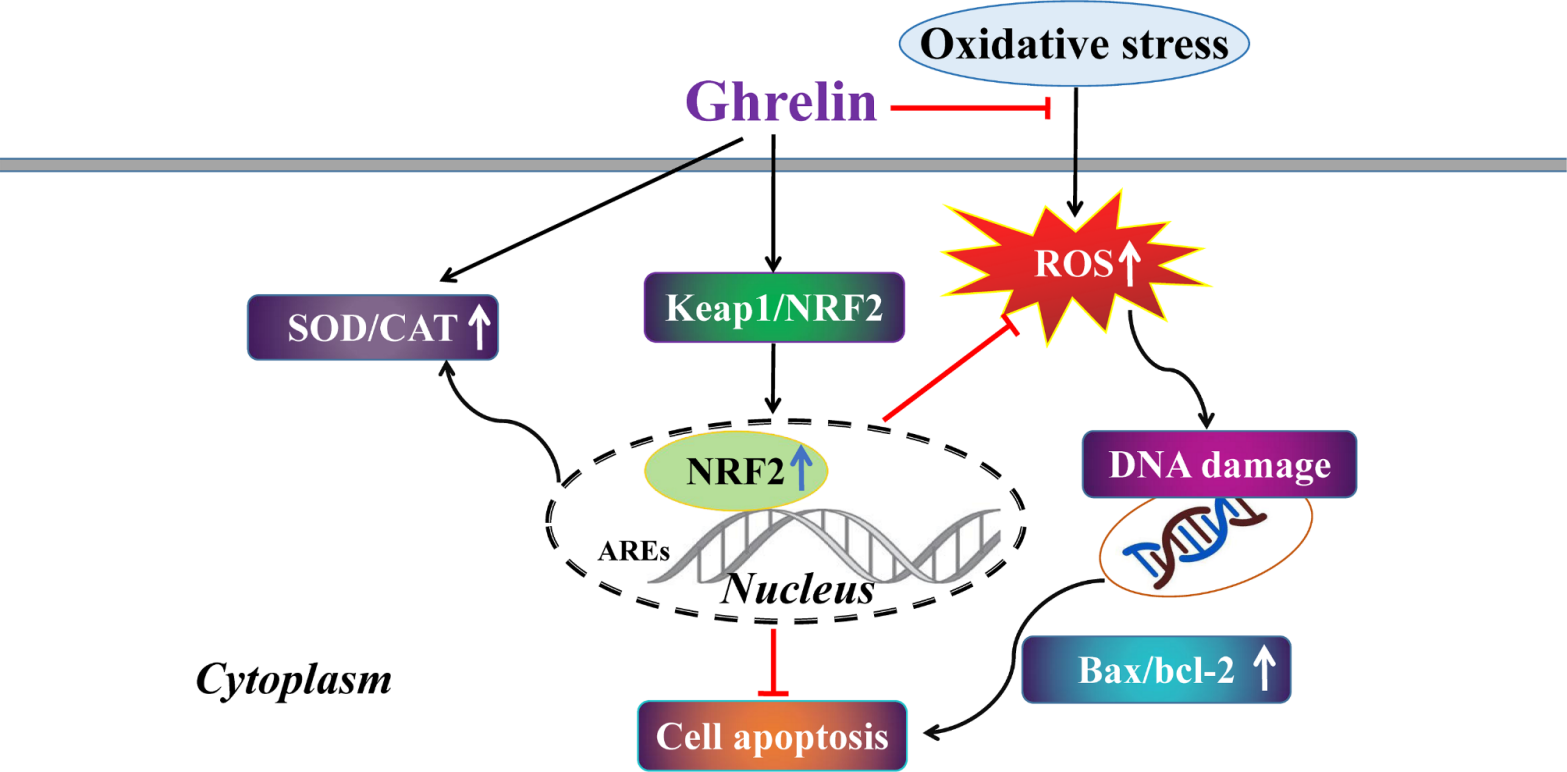
Summary of the effects of ghrelin on H_2_O_2_-induced oxidative damage in ARPE-19 cells

Oxidative stress is the key factor in AMD. NaIO_3_ induces retinal injury through oxidative damage (Liu Yang et al. 2019). In this study, we examined the histology and morphology of rat retinal tissue. As shown in the results, ghrelin treatment had therapeutic effects on the increase in retinal thickness and reduction of apoptosis in retinal cells. These results suggest that ghrelin could promote the survival of retinal cells after oxidative stress, and inhibit apoptosis by regulating the expression of Bcl-2 and Bax (Fig. 5).

In summary, the present study elucidated the underlying mechanism of ghrelin alleviation in ARPE-19 cells and retinal damage. It will be helpful to provide a potential clinical in the clinic for retinal degenerative disorders, such as AMD.

## Data Availability

The data sets used and/or analysed during the current study are available from the corresponding author on reasonable request.
